# Fast and Efficient Image Encryption Algorithm Based on Modular Addition and SPD

**DOI:** 10.3390/e22010112

**Published:** 2020-01-16

**Authors:** Khushbu Khalid Butt, Guohui Li, Sajid Khan, Sohaib Manzoor

**Affiliations:** 1 School of Computer Science and Technology, Huazhong University of Science and Technology, Wuhan 430074, China; khushbukhalid@hust.edu.cn (K.K.B.); khansajid@hust.edu.cn (S.K.); 2 Faculty of Computer Science Department, Huazhong University of Science and Technology, Wuhan 430074, China; 3 School of Electronic Information and Communications, Huazhong University of Science and Technology, Wuhan 430074, China; sohaibmanzoor@hust.edu.cn

**Keywords:** image encryption, modular addition, scrambling plus diffusion (SPD), SHA-512, security, entropy

## Abstract

Bit-level and pixel-level methods are two classifications for image encryption, which describe the smallest processing elements manipulated in diffusion and permutation respectively. Most pixel-level permutation methods merely alter the positions of pixels, resulting in similar histograms for the original and permuted images. Bit-level permutation methods, however, have the ability to change the histogram of the image, but are usually not preferred due to their time-consuming nature, which is owed to bit-level computation, unlike that of other permutation techniques. In this paper, we introduce a new image encryption algorithm which uses binary bit-plane scrambling and an SPD diffusion technique for the bit-planes of a plain image, based on a card game trick. Integer values of the hexadecimal key SHA-512 are also used, along with the adaptive block-based modular addition of pixels to encrypt the images. To prove the first-rate encryption performance of our proposed algorithm, security analyses are provided in this paper. Simulations and other results confirmed the robustness of the proposed image encryption algorithm against many well-known attacks; in particular, brute-force attacks, known/chosen plain text attacks, occlusion attacks, differential attacks, and gray value difference attacks, among others.

## 1. Introduction

### 1.1. Background

Recent technological advancements in networks and communication technologies have significantly improved communications. As a result, multimedia security (with respect to the communication of multimedia content) became of widespread concern, especially when the spread of multimedia content became out of control due to almost global internet access. Thus, the public is at a serious risk of confidential information leakage and has become more concerned about the security of multimedia communication in the modern, globalized world [1].

Internet technology has progressed greatly and digital image processing techniques have improved along with it, resulting in the availability of various applications for digital images. Such applications are expected to increase in leaps and bounds in the near future. Recently, image security has attracted considerable attention, as trillions of images are transmitted over the internet every day—shared through mobile phones and other digital devices, and outsourced to cloud storage. This has led to the rise of encryption as the most popular technique for protecting the privacy of users and their digital images. Various image encryption algorithms have been developed, which have utilized different kinds of techniques for this purpose [2,3,4,5,6,7,8].

Over the last decade, a variety of chaotic systems have been developed [9,10] for the construction of image cryptosystems, including and not limited to the 3D baker map [11], the 2D logistic map [12], the 3D cat map [13], the 2D standard map [14], the 3D LCA map [15], and the 2D henon map [16]. Moreover, some chaotic systems have been combined with other approaches for this purpose, such as DNA computing [17,18], one-time keys [19], cellular automata [20], and the perceptron model [21], which have been used for the specific objective of enhancing image cryptosystem security.

There are two main stages in chaos-based image encryption systems, with respect to their composition: permutation and diffusion. The repetition of these two stages multiple times can attain a desirable security level. The first stage (called the permutation stage) tends to decrease strong relationships between pixels that are adjacently located. Permutation methods can be classified into two further categories: bit-level and pixel-level, corresponding to the smallest processing entity in each case.

In the first category, a pixel is considered the smallest scrambling element in permutation; for example, a new block image encryption algorithm that was proposed by Wang et al. [22] used a random grouping technique to scramble the image pixels with the help of the Arnold cat map. This particular method generally eliminated the drawback of periodicity of the Arnold cat map. Another method, proposed by Hua et al. [23] known as the high-speed image scrambling method, changes the positions of rows and columns of pixels at the same time, thereby efficiently reducing strongly correlated neighboring pixels.

Parvin et al. [24] came up with and implemented a new strategy for scrambling images by shifting the pixels in rows and columns through a chaotic sequence. Their strategy is very easy to implement, along with being very efficient. Usually, pixel-based permutation uses the strategy of shuffling the image by changing pixel positions without having to modify the pixel values; thus, the histograms of the permuted images and the original images tend to be identical, in most cases, making these pixel-level based permutations susceptible to histogram attacks and known or chosen plain text attacks, if these images do not go through diffusion or have bad diffusions [25,26].

A bit is regarded as the building block and basic operating element in the second category of permutation. For this purpose, the plain image’s pixel matrix is usually transformed into a binary matrix. For example, a discriminatory image encryption system based on bit-level permutation was proposed by Tao et al. [27], as each bit tends to contribute different information to an image under consideration of encrypting the first four most significant bits of each of pixel and leaving the remaining (i.e., least significant) four bits unaffected. Some other researchers [28,29] have proposed other improved schemes, in which they applied two distinct methods for permuting the most significant four bits and least significant four bits.

Liu and Wang [9] pointed out some weaknesses in these improved schemes [30], such as the schemes requiring a square original image for encryption and that they are able to permute these bits merely inside each bit-plane. High dimensional chaotic maps and bit-level permutation has also been proposed by Liu et al. for encrypting color images, but these methods tend to take more computational time than pixel-based scrambling techniques.

The fundamental characteristics of bit distribution have been analyzed by Zhang et al. [31], who proposed a multiplication and shrink scheme in the permutation phase to reduce the high correlation and dependency among significant bits and to make the distribution for each bit-plane smooth. However, such basic image characteristics at bit-level, as proposed by Zhang, have been regarded to be inapplicable to medical-related images by Chen et al. [32]. Uniform bit distribution, along with certain different image diffusion performance, could also be achieved at the same time in the permutation stage by use of a non-linear inter-pixel computing and substitution methodology.

Apart from chaos theory, various other techniques have been used to design image ciphers [33,34,35]. Some examples of such techniques include an image encryption scheme developed by the authors that utilizes Latin sequences for pixel permutation and substitution [36], and an innovative image encryption technique that uses a gray code for pixel permutation and a plain pixel diffusion structure for achieving the diffusion property [37].

A new hybrid encryption scheme based on FSM and cellular automata, which accompanied a DNA sequence, has also been introduced by Sajid et al. [38]. Their particular scheme provided good results, along with the concept of creating and using a local rule regarding algorithm efficiency; however, their particular technique had limitations, as its usage was confined to gray-scale images.

### 1.2. Contributions

After analysis of the above-mentioned research, we found that the complexity of the particular encryption technique and number of confusion–diffusion rounds are the main reasons for the high encryption times of some of the earlier image encryption algorithms. Thus, to address the problems of high complexity and encryption time in the above-mentioned algorithms, we propose a new simple, fast, and efficient image encryption algorithm, based on segmentation of the image into m×n blocks, modular addition between the pixels, and a novel method for pixel diffusion. Together, these steps increase the security level of the ciphered images against well-known cryptanalysis attacks. We have made three major contributions with this model:

First, adaptive block-based modular pixel addition is performed. In every 4×4 block, the pixels are diffused by modular addition in such a way that, for every forthcoming block (n), the pixels of the former block (*n*−1) are added with integer values of a hexadecimal key entity to form a direct relationship between the previous block and the upcoming block.

Second, a card trick-based scrambling plus diffusion (SPD) method is proposed. For every channel, the key bit-plane can be the same or different, which can be chosen. The SPD method efficiently diffuses the pixels and reduces the correlation among the pixels, thereby enhancing the security of the key.

Third, the SHA-512 hexadecimal key (K) value is used for both modular addition and generating a seed value for the random matrix. Depending on the image size, the integer values of hexadecimal key pairs can be used in repetition or only once by taking a huge hexadecimal key (i.e., the key can be “K ≥M×N” the size of an image). This also provides us a choice of whether to enlarge the key length. The rest of the paper is organized as follows: in Section 2, the preliminary work is presented. In Section 3, the proposed image encryption scheme is detailed. Our simulations and discussion are presented in Section 4, and our security analysis is in Section 5. Finally, our conclusions are given in Section 6.

## 2. Preliminary Work

Every decimal number can be denoted by a binary number. As an image matrix is in decimal matrix form, a binary sequence {$bin_{\left(0\right)}+bin_{\left(1\right)}+,\ldots ,+bin_{\left(l-2\right)}+bin_{\left(l-1\right)}$} can be used to represent a particular image in the following way:

(1) $$DN=\sum_{p=0}^{l-1}bin_{\left(p\right)}2^{p}=bin_{\left(0\right)}2^{0}+bin_{\left(1\right)}2^{1}+\ldots +bin_{\left(l-1\right)}2^{l-1}.$$

As an RGB image is comprised of three channels, each having decimal values between 0–255, its pixels can also be denoted by a binary sequence. By doing so, the RGB image encompasses 8 bit-planes for each channel, for a total of 24 bit-planes for the image [39]. In bit-plane representation form, the *i*th bit-plane contains all the *i*th binary values of every pixel in the particular image.

### 2.1. Scrambling Plus Diffusion (SPD)

An effective practice to diminish the correlation between neighboring pixels is scrambling. In this paper, we introduce a novel method for bit-plane scrambling plus diffusion (SPD). In the proposed method, bit-planes are scrambled, but at the same time, the pixels values are diffused by altering the binary sequence of every pixel; hence, the technique is called SPD. The proposed novel method of SPD is based on a card trick. A video of this trick can be seen in the following link: (https://www.youtube.com/watch?v=2E-FNqICDgg). The scrambling is comprised of two phases.

First, before performing the SPD and modular addition, simple scrambling is performed over the plain image to shuffle the plain pixel values. The code for ordinary scrambling can be found in the Supplementary Materials. Second, the proposed SPD method is basically designed for 8 bit-planes, but, because in a color image there are three channels and each channel has 8 bit-planes for a total of 24 bit-planes, we have a choice to either use the same key bit-plane for all three channels or different keys to enlarge the key size and make it stronger. In the procedure below, instead of elaborating a lengthy sequence combination for all 24 bit-planes, we explain the process for just one channel. First of all, we map the 8 bit-planes of a particular channel of the image with the eight variables given below:

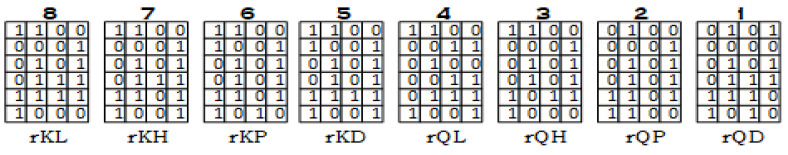



If the key bit-plane is **KL**, the arrangement will be as follows.

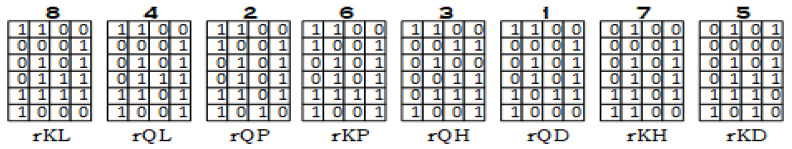



If the key bit-plane is **KH**, the arrangement will be as follows.

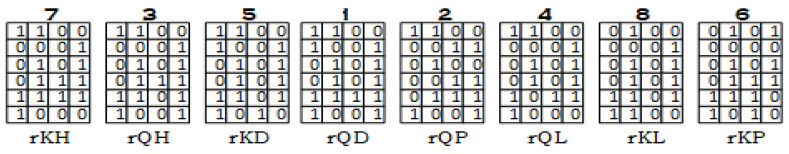



If the key bit-plane is **KP**, the arrangement will be as follows.

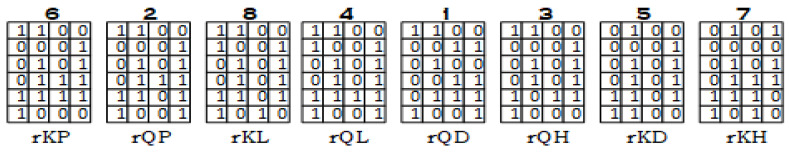



If the key bit-plane is **KD**, the arrangement will be as follows.

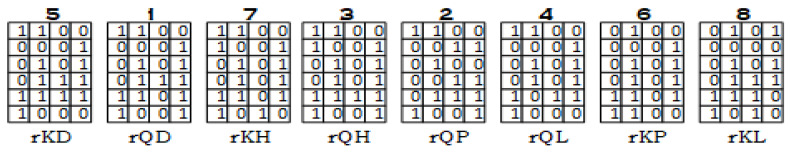



If the key bit-plane is **QL**, the arrangement will be as follows.

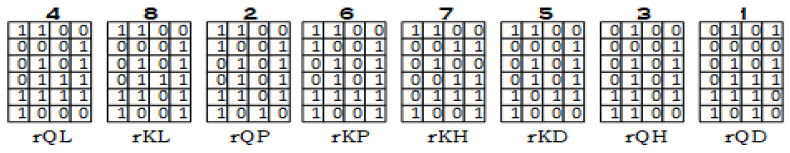



If the key bit-plane is **QH**, the arrangement will be as follows.

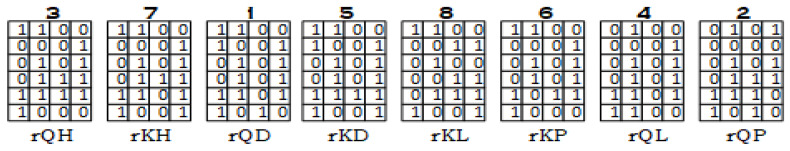



If the key bit-plane is **QP**, the arrangement will be as follows.

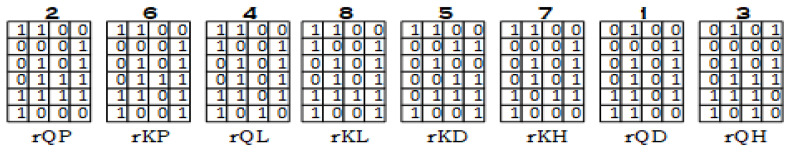



If the key bit-plane is **QD**, the arrangement will be as follows.

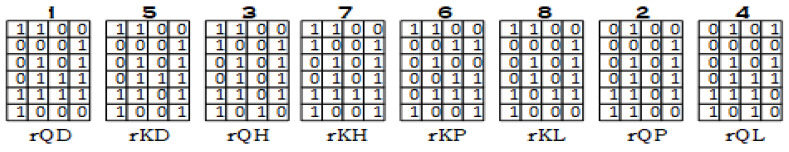



## 3. Image Encryption

**Figure 1:**
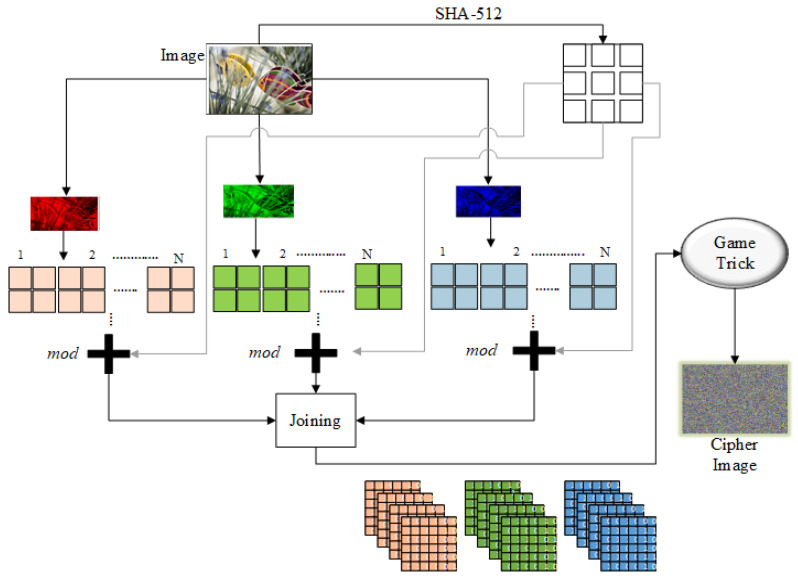
General block diagram.

The general structural diagram of our proposed algorithm is given in Figure 1. The proposed image encryption is comprised of two stages: the first is modular addition and the second is SPD over the binary bit-planes for each of the RGB channels. The detailed encryption steps are discussed below.

**Step 1**: Obtain an image of size H×W and take the SHA-512 to get its 128 bit hexadecimal key; arrange the hexadecimal key, and get the decimal value of each key entity.

**Step 2**: Take the seed value from the hash key value to generate a random matrix equal in size to the input image (H×W). Perform the scrambling operation.

**Step 3**: Divide the scrambled image into RGB channels, as shown below:

$$\begin{array}{c} Image_{RGB}↔\left\{\begin{array}{c} RedChannel.\\ GreenChannel.\\ BlueChannel. \end{array}\right. \end{array}$$

**Step 4**: Subdivide each channel, and the random matrix, into 4×4 blocks as follows:

(2) $$Red=B_{r1},B_{r2},B_{r3},\ldots ,B_{r(n-1)},B_{rn}$$

(3) $$Green=B_{g1},B_{g2},B_{g3},\ldots ,B_{g(n-1)},B_{gn}$$

(4) $$Blue=B_{b1},B_{b2},B_{b3},\ldots ,B_{b(n-1)},B_{bn}$$

(5) $$Random_{matrix}=B_{RM1},B_{RM2},B_{RM3},\ldots ,B_{RM(n-1)},B_{RMn},$$

where $B_{i}=\left(1,2,3,\cdots ,n\right)$ represents the particular block; *r*, *g*, and *b* represent the red, green, and blue channels, respectively; $B_{RM(i)}=\left(1,2,3,\cdots ,n\right)$ represents the particular random matrix block; and *n* is the total number of blocks in the particular image.

**Step 5**: Take the red channel and perform modular addition over the pixels in every block to encrypt the blocks, as described below:

(6) $$\begin{array}{c} Red=\left\{\begin{array}{c} E_{r1}=\left[B_{RM1}+Br_{1}+IN_{k1}\right]mod256\\ E_{r2}=\left[B_{RM2}+Br_{2}+E_{r1}+IN_{k2}\right]mod256\\ E_{r3}=\left[B_{RM3}+Br_{3}+E_{r2}+IN_{k3}\right]mod256\\ .\\ .\\ .\\ E_{r(n-1)}=\left[B_{RM(n-1)}+Br_{\left(n-1\right)}+E_{r(n-2)}+IN_{k(n-1)}\right]mod256\\ E_{rn}=\left[B_{RM(n)}+Br_{n}+E_{r(n-1)}+IN_{kn}\right]mod256, \end{array}\right. \end{array}$$

where $E_{rn}$ represents an encrypted pixel of the red channel block, $IN_{kn}$ represents the integer value of a particular hexadecimal key entity, and mod is the modular function, which returns a pixel value between 0 and 255.

**Step 6**: For the green channel, repeat Step 5 over all blocks to encrypt the green blocks, as described below:

(7) $$\begin{array}{c} Green=\left\{\begin{array}{c} E_{g1}=\left[B_{RM1}+Bg_{1}+IN_{k1}\right]mod256\\ E_{g2}=\left[B_{RM2}+Bg_{2}+E_{g1}+IN_{k2}\right]mod256\\ E_{g3}=\left[B_{RM3}+Bg_{3}+E_{g2}+IN_{k3}\right]mod256\\ .\\ .\\ E_{g(n-1)}=\left[RM_{\left(n-1\right)}+Br_{\left(n-1\right)}+E_{g(n-2)}+IN_{k(n-1)}\right]mod256\\ E_{gn}=\left[B_{RM(n)}+Br_{n}+E_{g(n-1)}+IN_{kn}\right]mod256. \end{array}\right. \end{array}$$

Perform the modular addition on the blue channel to encrypt its pixels, as described in Step 5. In Equations (6) and (7) in each step, sum the original block, random block, key, and previous encrypted block resemble the cipher block chaining mode [40].

**Step 7**: Join all the encrypted sub-blocks to obtain an image matrix and divide each channel into binary bit-planes, as given below:

(8) $$\begin{array}{c} Image_{RGB}=\left\{\begin{array}{c} Red_{i,j}=Bin_{r1},Bin_{r2},Bin_{r3},\ldots ,Bin_{r8}\\ Green_{i,j}=Bin_{g1},Bin_{g2},Bin_{g3},\ldots ,Bin_{g8}\\ Blue_{i,j}=Bin_{b1},Bin_{b2},Bin_{b3},\ldots ,Bin_{b8}, \end{array}\right. \end{array}$$

where $Bin_{ij}$ represents the *i*th bit window of particular channel.

**Step 8**: Select the key bit-planes (i.e., KL, QL, KP, and so on) for each of the RGB channels and perform bit-plane scrambling, as described in Section 2.1.

**Step 9**: Join all the bit-planes to the respective sequences to obtain the cipher image:

(9) $$Cipher_{\left(M,N,3\right)}=C_{\left(i,j\right)}^{red};C_{\left(i,j\right)}^{green};C_{\left(i,j\right)}^{blue},$$

where *M* and *N* represent the height and width of the cipher image.

## 4. Simulation Results and Discussion

Simulation was carried out using the JetBrains PyCharm Edu 2019.1.1 × 64 software installed on a PC with 4 GB memory, an Intel Core I5 Processor, and the Windows 10 Enterprise operating system. For the histograms, Matlab R2017a was used.

**Figure 2:**
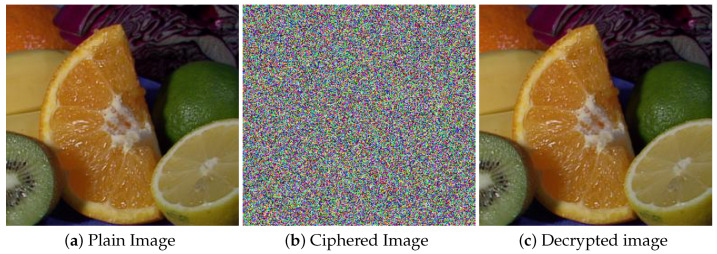
Encryption and decryption results. (**a**) Plain image of fruits; (**b**) cipher image of fruits; (**c**) decrypted image of fruits.

**Figure 3:**
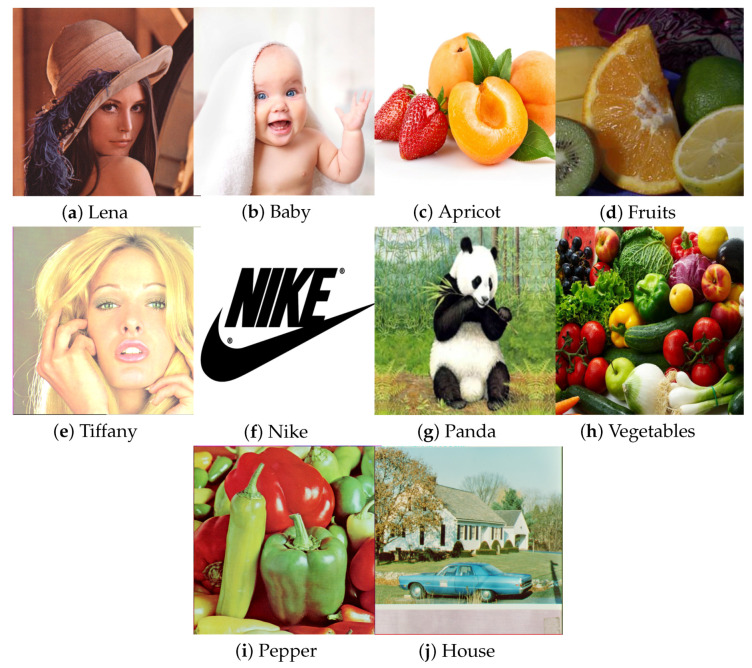
All test images.

The main test image (fruits), along with its cipher and decrypted image, are displayed in Figure 2, while all the test images that we used are shown in Figure 3. The key and other experimental parameters are given in Table 1.

Before performing decryption, the secret key needs to be transferred to the receiver through a secure channel, and the three key bit-planes for the red, green, and blue channels. In decryption, all steps are performed in the reverse direction, while the modular addition phase equation in the decryption process is as follows:

(10) $$\begin{array}{c} Channel_{R,G,B}=\left\{\begin{array}{c} PI_{c(1)}=\left[E_{c(1)}-B_{RM1}-IN_{k1}\right]mod256\\ PI_{c(2)}=\left[E_{c(2)}-B_{RM2}-PI_{c(1)}-IN_{k2}\right]mod256\\ PI_{c(3)}=\left[E_{c(3)}-B_{RM3}-PI_{c(2)}-IN_{k3}\right]mod256\\ .\\ .\\ .\\ PI_{c(n-1)}=\left[E_{c(n-1)}-B_{RM(n-1)}-PI_{c(n-2)}-IN_{k(n-1)}\right]mod256\\ PI_{c(n)}=\left[E_{c(n)}-B_{RM(n)}-PI_{c(n-1)}-IN_{kn}\right]mod256, \end{array}\right. \end{array}$$

where $PI_{c(i)}$ represents the plain image blocks of the channel c∈R,G,B and $E_{c(i)}$ represents an encrypted block of the particular channel.

## 5. Security Analysis and Tests

### 5.1. Key Space and Key Sensitivity

First, the key space of an encryption algorithm should be appropriate. In the proposed algorithm, we use SHA-512, which gives an output of 512 bits in hexadecimal form. In SPD, three key bit-planes and their resultant possible combinations, and the long seed value of the hexadecimal key for the random matrix, can be included in the key space. Together, these form the key space of the proposed algorithm; $2^{512}$ means $1.340\times 10^{154}$. Thus, it has apt size and great capacity for protecting from brute-force attacks [41]. Secondly, the key must be tremendously sensitive to bit changes. If the key is not subtle enough, then a similar key may also be able to decrypt the cipher image [42,43].

Thus, to test the key sensitivity, we checked the number of bit change rate (NBCR) [44]. The NBCRs of the two images *B*_1_ and *B*_2_ are defined as

(11) $$NBCR\left(B_{1},B_{2}\right)=\frac{ham(B_{1},B_{2})}{T_{b}},$$

where ham $\left(B_{1},B_{2}\right)$ indicates the Hamming distance between $B_{1}$ and $B_{2}$, whereas $T_{b}$ is the overall number of bits from $B_{1}$ or $B_{2}$. If the acquired NBCR is near to 50%, then $B_{1}$ and $B_{2}$ are entirely dissimilar images, with no relationship.

$$K_{1}=E9BAD688008A995B2..\ldots ...B39ADC292.$$

For the key sensitivity of the proposed algorithm, our experiments were designed as follows:

1st: Alter one bit of *K*_1_ to obtain *K*_2_.

2nd: Using $K_{1}$ and $K_{2}$, encrypt the plain-image *P* and generate two cipher-images $C_{1}$ and $C_{2}$. Then, compute their NBCR.

3rd: Decrypt the particular cipher-image $C_{1}$ by using $K_{1}$ and $K_{2}$ to get two decrypted images $D_{1}$ and $D_{2}$. Then, compute the NBCR.

**Figure 4:**
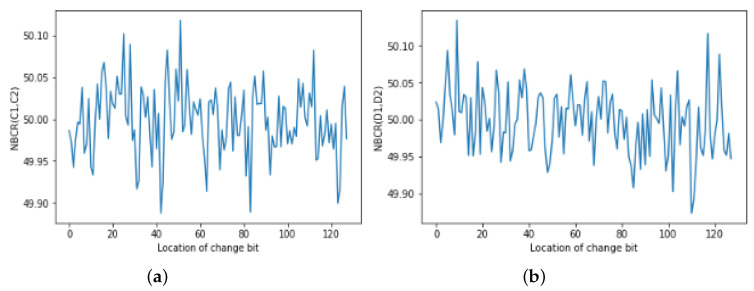
Key sensitivity analysis. (**a**) Number of bit change rate (NBCR) between C1 and C2; (**b**) NBCR between D1 and D2.

Figure 4 demonstrates the key sensitivity exploration result of the secret key for the fruit test image. We can observe from the graph that any change in the secret key will result in obtaining two cipher-images in the encryption phase and the two decrypted results in the decryption phase in entirely different forms. This shows that our proposed algorithm is extremely sensitive to deviation of the cipher key.

Furthermore, if the encryption algorithm has a less sensitive key, then any insignificant change in the real secret key will also lead to proper decryption of the actual image. Thus, a secret key of algorithm may seem corrupted, and as a result, the real key space will be smaller than the theoretical one [45,46].

Therefore, in order to further analyze the key sensitivity of our algorithm, we performed another experiment over the fruit image, in the encryption and decryption steps, by altering only one bit in the actual key, as shown below:

**Key_o_** = “E 9B AD 68 80 08 A9 95 B2 27 23 D5 56 54 11 F9 17 69 E9 E7 2F 64 07 B1 48 D8 4B 2D ED F1 DF 39 96 BF A2 75 EB E1 3B 9B 9C A2 7D 82 C6 44 07 64 A8 17 BD A1 A1 A5 4D E5 42 FD 42 7A B3 9A DC 29 2.”

**Key_1_** = “F 9B AD 68 80 08 A9 95 B2 27 23 D5 56 54 11 F9 17 69 E9 E7 2F 64 07 B1 48 D8 4B 2D ED F1 DF 39 96 BF A2 75 EB E1 3B 9B 9C A2 7D 82 C6 44 07 64 A8 17 BD A1 A1 A5 4D E5 42 FD 42 7A B3 9A DC 29 2.”

**Key_2_** = “E 9B AD 68 80 08 A9 95 B2 27 23 D5 56 54 11 F9 17 69 E9 E7 2F 64 07 B1 48 D8 4B 2D ED F1 DF 39 96 BF A2 75 EB E1 3B 9B 9C A2 7D 82 C6 44 07 64 A8 17 BD A1 A1 A5 4D E5 42 FD 42 7A B3 9A DC 29 3.”

**Key_o_** is the actual key, while **Key_1_** and **Key_2_** have only a one-bit key difference from the left-most side and the right-most side of the bit, respectively.

**Figure 5:**
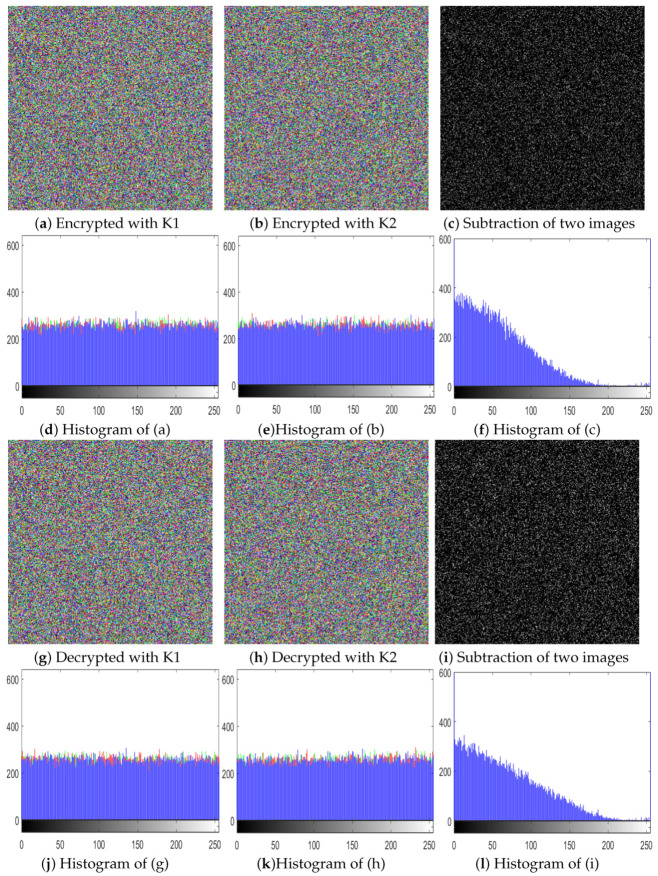
Key sensitivity test: (**a**) encrypted with K1; (**b**) encrypted with K2; (**c**) subtraction of (**a**,**b**); (**d**) histogram of (**a**); (**e**) histogram of (**b**); (**f**) histogram of (**c**); (**g**) decrypted with K1; (**h**) decrypted with K2; (**i**) subtraction of (**g**,**h**); (**j**) histogram of (**g**); (**k**) histogram of (**h**); (**l**) histogram of (**i**).

We performed experiments for both the encryption and decryption steps. In both cases, we selected keys with only a one-bit difference from the real key. $K_{1}$ is the key having a one-bit difference from the left side, while $K_{2}$ has a one-bit difference from the right side. Figure 5a,b shows the encrypted images with $K_{1}$ and $K_{2}$, respectively. Figure 5c is the subtraction result of (a) and (b). Figure 5d–f are the histograms of Figure 5a–c, respectively. Similarly, on the decryption side, we attempted to decrypt the actual cipher image with a key having one bit difference from the original key; the results are listed in Figure 5g,h. Figure 5i is the subtracted image of (g) and (h), and the respective histograms are shown in in Figure 5j–l. The results show that our proposed algorithm is highly sensitive to the key, and even a bit difference in the key will result in a totally noise-like image.

### 5.2. Histogram Analysis

**Figure 6:**
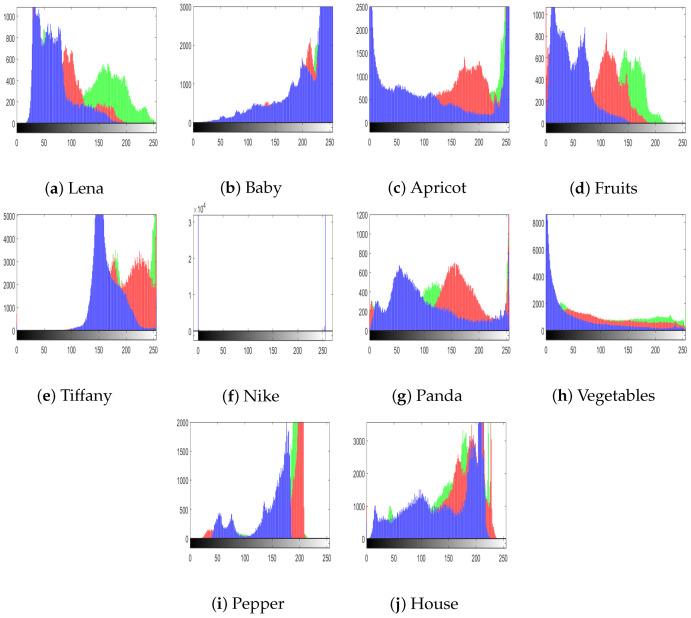
Histograms of plain test images.

**Figure 7:**
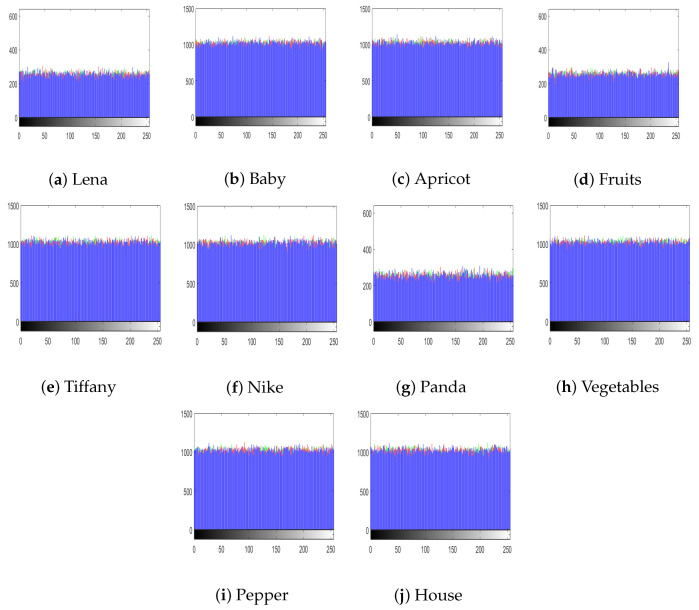
Histograms of cipher images.

**Figure 8:**
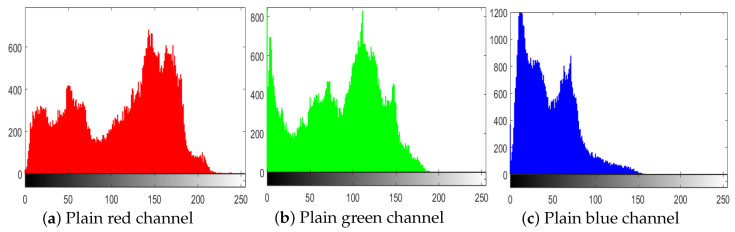

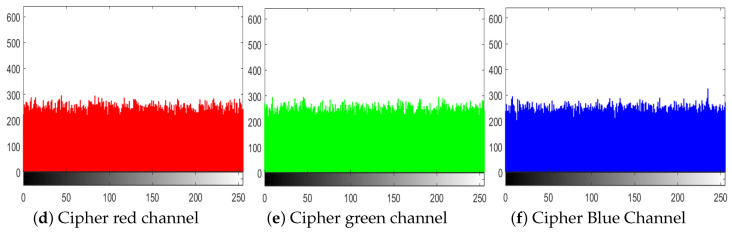
Histograms of RGB channels of fruits. (**a**) Plain red channel; (**b**) plain green channel; (**c**) plain blue channel; (**d**) ciphered red channel; (**e**) ciphered green channel; (**f**) ciphered blue channel.

The histograms of the images reveal the dissemination of the pixel values. Generally, an interloper can recuperate important information about the cipher image from its (inconsistent) histogram. Therefore, to thwart an impostor from recuperating useful information, it is essential that the histograms of images generated by the cipher show no numerical resemblance with the plain image; in addition, it must have an unvarying distribution of pixels. Figure 6a–j shows the histograms of all the test images that we used, and Figure 7 shows the histograms of corresponding cipher-generated images in the sequence of all test images. The histogram of main test image (fruit) is given in Figure 8. Figure 8a–c shows the histograms of the plain channels of the fruit image, and Figure 8d–f shows the histograms of the blue, green, and red channels, respectively, of the image encrypted by the proposed algorithm. It can be observed that the histograms of the cipher-generated images are nearly flat and uniform.

Likewise, by calculating the variances of the histograms, we assessed the consistency of the encrypted images. If the variance of an encrypted images is smaller, then the uniformity (and the security level) of the particular image encryption algorithm is greater [45,46].

(12) $$H_{variance}\left(W\right)=\frac{1}{n^{2}}\sum_{r=1}^{n}\sum_{c=1}^{n}\frac{1}{2}{\left(w_{r}-w_{c}\right)}^{2},$$

where $W=w_{1},w_{2},\cdots ,w_{256}$ represent the vectors of the histogram values, and $w_{r}$ and $w_{c}$ are pixels with gray values equal to *r* and *c*, correspondingly.

Table 2 lists the variances of some test images. From the table values, it can be concluded that the variances of the plain images were too high, but for the ciphered images, they were very low. For example, the average variance of the cipher image of Lena (256×256) was 229, but it was 81,231 for the plain Lena image. The given comparison also shows that for the majority of test images, the histogram variances of images encrypted by the proposed algorithm were lower than those in [41,43]. On the basis of this comparison, we can say that our proposed algorithm has the ability to make the encryption algorithm more secure.

### 5.3. Pixel Correlation Analysis

To check the relationships amongst neighboring pixels in the encrypted and plain images, a pixel correlation analysis test was performed. For an encryption algorithm, it is essential to diminish the correlation amongst neighboring pixels, in order to prevent the leakage of authentic information.

**Figure 9:**
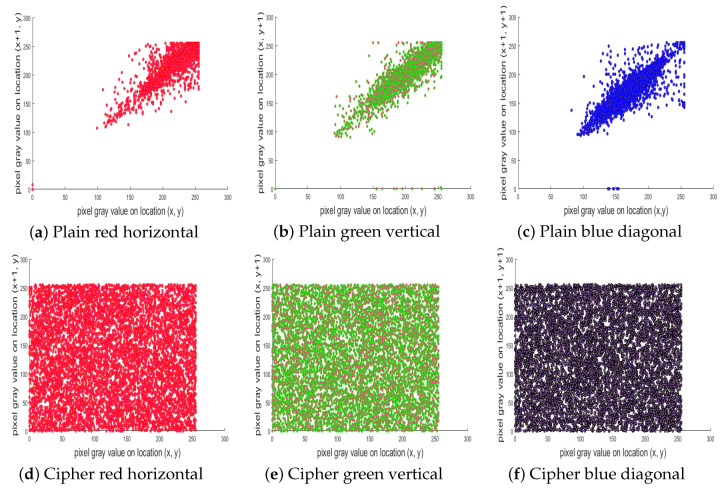
Pixel correlation of test image tree. (**a**) Plain red channel pixels, horizontal; (**b**) plain green channel pixels, vertical; (**c**) plain blue channel, diagonal; (**d**) ciphered red channel pixels, horizontal; (**e**) ciphered green channel pixels, vertical; (**f**) ciphered blue channel pixels, diagonal.

The correlation coefficient *C_orr._(i,j)* between the adjacent pixels of an image can be computed by the following equations:

(13) $$C\left(\hbar \right)=\frac{1}{L}\sum_{i=1}^{L}\hbar_{i}$$

(14) $$D\left(\hbar \right)=\frac{1}{L}\sum_{i=1}^{L}{\left(\hbar_{i}-E\left(\hbar \right)\right)}^{2}$$

(15) $$C_{var.}\left(\hbar ,w\right)=\frac{1}{L}\sum_{i=1}^{L}\left(\hbar_{i}-E\left(\hbar \right)\right)\left(w_{i}-E\left(w\right)\right)$$

(16) $$C_{r(\hbar ,w)}=\frac{C_{var.}\left(\hbar ,w\right)}{\sqrt{D(\hbar )\times D(w)}},$$

where (*ℏ, w*) is the gray value of neighboring pixels, *L* is the entire number of pixels that have been selected from an image, *C_var._(ℏ, w)* is the covariance, *D(w)* is the variance, and *E(ℏ)* and *E(w)* denote the expectation of the variables *ℏ* and *w*, respectively. Table 3 lists the correlations of 1000 pixels in the Lena image. The average value of the pixel correlation in the horizontal, vertical, and diagonal directions is −0.0092375. Comparison with earlier algorithms indicates that the correlation between neighboring pixels in our ciphered image is less than those in [47,48,49], while it was comparable to those of [50,51]. In Table 4, the pixel correlation values of the images of USC-SIPI database, as well as other test images, are given. From the table values, we can see that the correlation in the plain image is too high (almost equal to “1” for each channel), but in the images encrypted by our proposed algorithm, it is very low. This confirms the satisfactory security level of our algorithm. Figure 9a–f shows plots of the correlation of two neighboring pixels in the plain image “tree” and its cipher-generated image. The plot shows the red channel horizontally, the green channel vertically, and the blue channel diagonally distributed. These results also show that the correlation is highly reduced in the cipher image, and thus, we can say that attackers cannot obtain any information from the cipher image in this way.

### 5.4. Differential Attack

Usually, a cracker performs a little amendment to the pixels of the plain image and then uses the same encryption approach to encrypt similar images. With this approach, they try to find the association between a plain image and an encrypted image. To check the robustness of our proposed method against differential attacks, we employed the number of pixels change rate (NPCR) and unified average changing intensity (UACI). The NPCR and UACI can be computed by the following equations:

(17) $$NPCR_{C_{1},C_{2}}=\left[\frac{\sum_{i,j}\tilde{I}\left(i,j\right)}{H\times W}\right]\times 100$$

(18) $$UACI_{C_{1},C_{2}}=\frac{1}{H\times W}\left[\sum_{i,j}\frac{|C_{1}\left(i,j\right)-C_{2}\left(i,j\right)|}{2^{8}-1}\right]\times 100,$$

where *C_1_* and *C_2_* represent two dissimilar ciphered images, before and after one pixel in the plain image is altered, respectively; *H* and *W* are the height and width of the image; and $\tilde{I}\left(i,j\right)$ can be defined as follows:

(19) $$\tilde{I}\left(i,j\right)=\left\{\begin{array}{cc} 0 & C_{1}\left(i,j\right)\neq C_{2}\left(i,j\right)\\ 1 & otherwise \end{array}\right.$$

The NPCR and UACI values for two images (Lena, pepper) are given in Table 5, from which we can see that NPCR≥99.6 and UACI≥33.4. From these values, it can be seen that the NPCR values of the proposed encryption algorithm were better than those of [52,53,54,55], while the UACI value was comparable to the others.

### 5.5. Known and Chosen Plain Text Analysis

Generally, there are four types of cryptanalysis attacks which can be performed to break an image encryption algorithm: chosen-plain text attack, chosen-cipher text attack, known-plain text attack, and cipher text-only attack [58,59,60]. The cryptanalysis method where the attacker picks certain plain text to obtain the related cipher text is called chosen-plain text attack. By probing the plain text and the related cipher text, they can attempt to deduce some valuable encrypted information. Finally, by means of that information, they can try to convalesce the actual images [61,62].

**Figure 10:**
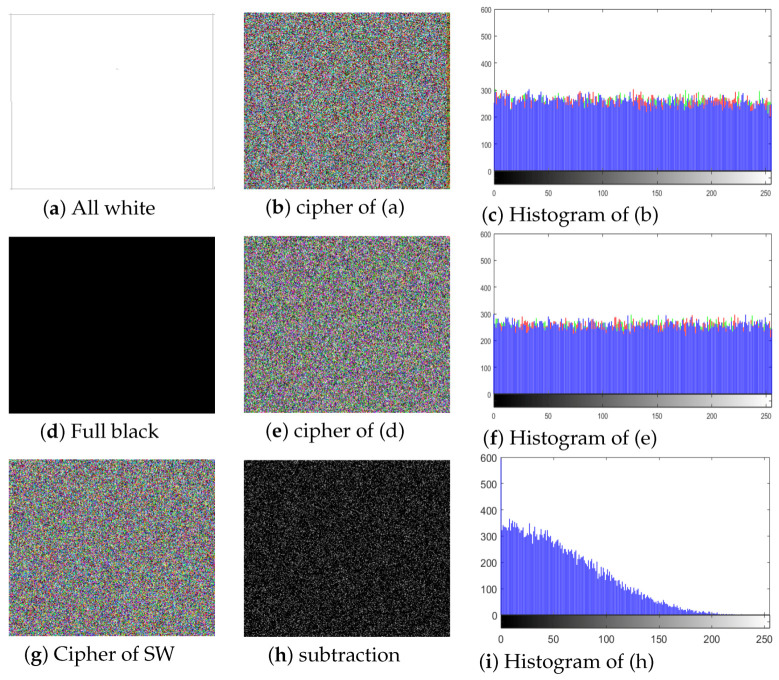
Encryption result of all white and full black images. (**a**) All white image; (**b**) full black image; (**c**) cipher image of (**a**); (**d**) cipher image of (**b**); (**e**) histogram of (**c**); (**f**) histogram of (**d**); (**g**) cipher of one pixel change of (**a**); (**h**) subtraction of (**b**,**g**); (**i**) histogram of (**g**).

Of the above-mentioned attacks, the chosen-plain text attack is the most influential, so if an encryption algorithm can successfully withstand this attack, it is believed to have a satisfactory security level against the other three attacks as well. We performed this attack by using all-white and full-black images. We also created special white (SW) and special black (SB) images, having all pixels 255 in the white image and 0 in the black image, except for one pixel at the location 200×200, which was different. We created the cipher images and the results are given in Figure 10. In Figure 10a,d, the full white and full black images are shown; Figure 10b,e are the corresponding cipher images, respectively. Additionally, Figure 10c,f shows the histograms of Figure 10b,e; Figure 10g is the cipher image of the special image; and Figure 10h is the subtraction result of the cipher of the all-white image and the cipher of the SW image. Figure 10i shows the histogram of the subtracted image. We can see that the histograms of the cipher-generated images of both the all-white and full-black images are almost uniform and flat, and that the subtracted image is also a noise-like image showing no useful information. In Table 6, the entropy, NPCR, UACI, and correlation values of the all-white, SW, and full-black images are also given. From the results, it is clear that the ciphered image of our proposed algorithm had higher entropy values, better correlation results (in comparison to [63]), and ideal NPCR and UACI values. On the basis of these results, we can conclude that our proposed algorithm has high security against known/chosen plain text attacks, and hence, it can keep images more secure.

### 5.6. Encryption Quality

The image encryption quality can be determined by the below equation, where $E_{\left(r,c\right)}$ and $I_{\left(r,c\right)}$ can be taken as the gray values of the image pixels in a grid $G_{\left(r,c\right)}$ in the cipher and the plain images, respectively, each having H×W pixels with *n* gray levels; $I_{\left(r,c\right)}$ and $E_{\left(r,c\right)}\in \left(0,1,\cdots ,n-1\right)$; $H_{n}\left(E\right)$ and $H_{n}\left(I\right)$ are the occurrences of the gray level *n* in the cipher and plain images, respectively; and $E_{Q}$ depicts the average change rate for each gray level *n*. The higher the *E_Q_* value, the more secure the image is. $E_{Q}$ can be computed by the following formula:

(20) $$E_{Q}=\sum_{n=0}^{255}\frac{{\left(H_{n}\left(E\right)-H_{n}\left(I\right)\right)}^{2}}{256}.$$

Table 7 lists the $E_{Q}$ values of USC-SIPI database images, along with their comparison to [64]. The table values show that the $E_{Q}$ values of the proposed algorithm are higher that those of [64] in the majority of images. Thus, we can say that the proposed algorithm provides a more secure encryption algorithm.

### 5.7. Robustness Against Occlusion Attack

In image processing, the extensively used parameters to check the encryption quality are peak signal to noise ratio (PSNR) and mean square error (MSE). The PSNR and MSE values between the plain (P), decrypted (D), and ciphered images can be computed as follows:

(21) $$PSNR=10\times log_{10}(\frac{{\left(2^{L}-1\right)}^{2}}{MSE}\left(dB\right),$$

where *L* is the bit-depth of the particular image. The MSE can be defined as

(22) $$MSE=\frac{1}{HW}\sum_{r=1}^{H}\sum_{c=1}^{W}{\left[P_{\left(r,c\right)}-D_{\left(r,c\right)}\right]}^{2}.$$

**Figure 11:**
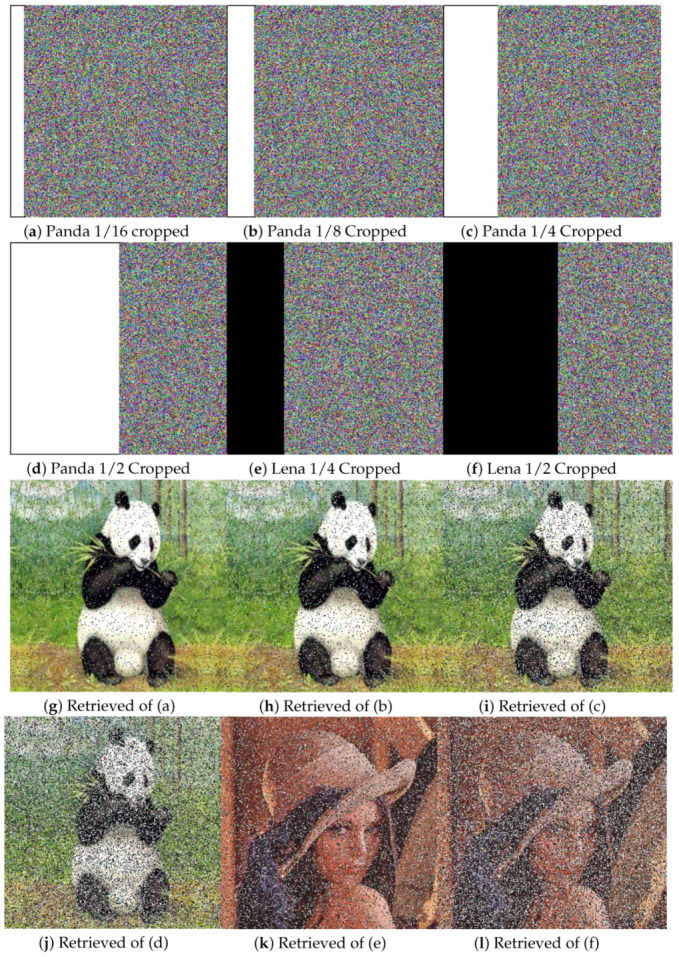
Occlusion attack test. (**a**) Cropped image of Lena; (**b**) cropped image of a panda; (**c**) cropped image of a house; (**d**) cropped image of baboon; (**e**) cropped image of a panda; (**f**) cropped image of Lena; (**g**) retrieved image of (**a**); (**h**) retrieved image of (**b**); (**i**) retrieved image of (**c**); (**j**) retrieved image of (**d**); (**k**) retrieved image of (**e**); (**l**) retrieved image of (**f**).

A higher PSNR value indicates a smaller difference between the plain and decrypted images. If P and D are the same, their PSNR will be infinity. Table 8 lists the PSNR values between the plain and ciphered images and plain and decrypted images for the different test images, along with comparisons to other methods. The infinity value of PSNR for plain to decrypted shows that the proposed algorithm can efficiently decrypt the actual image, while the method of [65] resulted in loss of some information. Similarly, lower values of PSNR between the plain and cipher images indicate more secure image encryption, as lower values indicate greater differences. The lower PSNR values of plain to ciphered images in Table 8 indicate that the proposed algorithm encrypts better than those of [65,66,67]. An alternative means of quality checking is at the receiver side, where a ciphered image may lose some data or get blurred. Thus, a reliable encryption algorithm or method should be able to get back the actual image without dropping too much substantial information. Figure 11a–d shows the test image Panda with different cropped portions (i.e., 1/16, 1/8, 1/4, and 1/2, respectively), while Figure 11g–j shows the images retrieved from the respective cropped image. Similarly, Figure 11e,f shows the Lena image cropped by 1/4 and 1/2, respectively, with the corresponding retrieved images in Figure 11k,l, respectively. We can see that retrieved image still has visual information, even after half of the ciphered image is destroyed. Table 9 lists a comparison of the PSNR and MSE of the Lena image with different cropped portions. The higher PSNR values and lower MSE values, as compared to [64,68], prove that proposed algorithm can retrieve images without losing substantial information.

### 5.8. Local and Shannon Information Entropy

Entropy (E) values indicate the degree of chaos in an encryption system by calculating its gray value probability. Information entropy (IE) or entropy can be defined in the following way:

Suppose an information source is *ℏ*. Then, *E* can be calculated with the following formula:

(23) $$E\left(\hbar \right)=\sum_{i=0}^{2^{l}-1}\rho \left(\hbar_{i}\right)log_{2}\frac{1}{\rho (\hbar_{i})}.$$

In the above equation, *l* depicts the gray intensity level and $\rho (\hbar_{i})$ represents the probability of the symbol $\hbar_{i}$. For an image with an intensity level of 2^8^, the ideal value of *E* is 8 [61]. Therefore, the closer the entropy is to this value, the better the randomness of the image pixels. A higher value ensures that less information will be discovered from the particular encrypted image. Table 10 shows the entropy value of our test images, along with a comparison to some existing image encryption algorithms. The values in the table show that *E* ≥ 7.996, which is higher than those of [10,58,62,70,71].

In [72], a new uncertainty test was proposed for native image blocks by means of the Shannon entropy. The Shannon entropy for native image pixel blocks (τ, ${\vec{a}}_{k}$) can be computed in following way:

(1): Randomly choose (non-overlapping) blocks in an image (i.e., $N_{1},N_{2},N_{3},\ldots ,N_{k}$) with ${\vec{a}}_{k}$ pixels within the image *I* having intensity $L^{n}$. (2): Calculate the Shannon entropy for all i∈((k−1),(k−2),…,3,2,1) using Equation (23). (3): Compute the Shannon entropy by sample mean over all *k* image pixel blocks $N_{1},N_{2},N_{3},\ldots ,N_{k}$ with the following formula:

(24) $$\hbar_{\left(\tau ,{\vec{a}}_{k}\right)}\left(N\right)=\sum_{i=1}^{k}\frac{H(N_{i})}{k}.$$

We calculated the Shannon entropy values for the ciphered images with k=32 and ${\vec{a}}_{k}$ = 1940 pixels. Table 11 lists the global and Shannon entropy values for the USC-SIPI image database, and some well-known test images. The values were E≥7.998, which is equivalent to the ideal value; furthermore, the Shannon entropy values were between 7.9018 and 7.9034. These results are comparable with those of [73].

### 5.9. Gray Value Degree (GVD) Analysis

GVD analysis is a well-known statistical test to check the randomness of an image. It can be calculated by comparing the cipher-generated image of a particular algorithm with the corresponding plain image. The ideal value is **1**, and the higher the value, the better the haphazardness and security. The GVD can be calculated using the following Equation:

(25) $$GVD\left(i,j\right)=\sum [\Gamma \left(i,i\right)-\Gamma \left(\overset{´}{i},\overset{´}{j}\right)],$$

where Γ(i,j) symbolizes the gray score at position (i,j), and $\left(\overset{´}{i},\overset{´}{j}\right)$ is as given below:

(26) $$\left(\overset{´}{i},\overset{´}{j}\right)=\left\{\begin{array}{c} \left(i,j+1\right)\\ \left(i,j-1\right)\\ \left(i+1,j\right)\\ (i-1,j). \end{array}\right.$$

The average neighborhood gray difference of an image can be calculated as follows:

(27) $${\left[GVD\left(i,j\right)\right]}_{avg.}=\frac{\sum_{i=2}^{M-1}\sum_{j=2}^{N-1}GVD\left(i,j\right)}{\left(M-2)(N-2\right)}$$

(28) $$GVD_{avg.}=\frac{\overset{´}{\chi }\left[G_{D}\left(r,c\right)\right]-\chi \left[G_{D}\left(r,c\right)\right]}{\overset{´}{\chi }\left[G_{D}\left(r,c\right)\right]+\chi \left[G_{D}\left(r,c\right)\right]}.$$

In the above equations, *M* and *N* represent the rows and columns of the image, respectively, and $\overset{´}{\chi }$ and χ symbolize the average score of the neighborhood gray value, where $\overset{´}{\chi }$ denotes after encrypting the image and χ denotes before encrypting the image. The final value is termed the GVD, which is **1** for completely dissimilar images but **0** for identical images. The GVD scores for the plain images of the **USC-SIPI** database and their cipher-generated images by the proposed algorithm are given in Table 12, along with comparisons to existing methods. In our case, the GVD scores indirectly varied with the key bit-planes (i.e., the GVD scores were higher when we selected the least significant bit plane as a key, and lower when we selected the most significant bit-planes as a secret key). The listed comparison shows that the GVD scores of the proposed algorithm were comparable those of [8,64].

### 5.10. Performance Comparison

**Figure 12:**
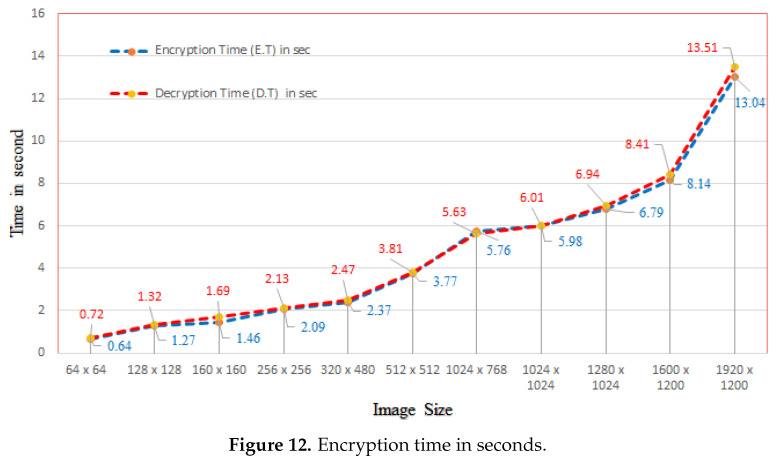
Encryption time in seconds.

Efficiency and time are key characteristics of an encryption algorithm. In chaotic, map-based image encryption schemes, the number of rounds for diffusion and permutation impact the encryption time. More rounds means a longer time taken by the algorithm to encrypt an image. As our proposed algorithm was based on modular addition, it took less time compared to many of the other algorithms listed in Table 13. Additionally, the encryption and decryption times (in seconds) for different-sized test images are shown in Figure 12.

## 6. Conclusions

To tackle the problems of complexity and encryption time, we have proposed a simple, fast, and secure image encryption algorithm which can ensure better security in less time, in comparison to some older algorithms. Our novel scrambling plus diffusion (SPD) technique is effective in two ways: it diffuses the pixels and scrambles the bit-planes in an efficient manner. The proposed algorithm can withstand many well-known attacks to ensure the security of the ciphered images. The use of modular addition between the pixels of image blocks and bit-plane scrambling provides an advantage in the sense of fast processing compared to binary addition between the pixels and pixel-based scrambling. Experimental results verified that our proposed algorithm can withstand some well-known attacks, such as key sensitivity tests, occlusion attacks, and known/chosen plain text attacks, and obtain satisfactory results from the GVD test. Therefore, we can conclude that the proposed encryption algorithm has demonstrated satisfactory security and efficiency. In further research, we will investigate its prospective applications in image communication.

## Figures and Tables

**Table 1:** Key and experimental parameters.

Terms	Key Parameters & Values
512-bit Hexa- decimal key	E9BAD688008A995B22723D5565411F91769E9E72F6407B148D84B2DEDF1DF3996BFA27 5EBE13B9B9CA27D82C6440764A817BDA1A1A54DE542FD427AB39ADC292
Seed Value	int(K,16) = 1224142479849059493616438967241173867216550529300133027919602126895 5249538595902639015802274554590625310127458155867404943493171251136512933387 374143849106
Integer values	int(K[0:1], 16) = 14, int(K[1:2], 16) = 9,..., int(K[(n-1):n], 16) = 2
Key bit-planes	Red${}_{QD}$; Green${}_{KH}$; Blue${}_{QL}$

**Table 2:** Histogram variance comparison.

Images	Plain			Proposed			[42] Cipher			[44] Cipher		
	Red	Green	Blue	Red	Green	Blue	Red	Green	Blue	Red	Green	Blue
Lena	123,072.500	87,100.835	33,522.734	219.513	231.052	221.119	247.78	279.62	265.71	527.32	504.75	501.68
Female	620,306.750	860,899.312	790,776.56	262.265	233.211	255.813	280.64	280.46	230.42	-	-	-
Couple	289,630.656	337,863.062	210,359.812	275.095	243.858	238.196	284.35	247.37	260.76	-	-	-
House	99,2034.125	1,330,180.125	768,126.75	1136.569	1119.742	1060.540	1070.2	1231.2	941.65	-	-	-
Tree	129,825.531	57,011.605	81,373.710	250.877	215.093	258.754	282.81	254.87	225.79	-	-	-
Bean	129,765.984	349,251.718	537,500.062	214.782	259.715	238.384	232.98	279.61	245.61	-	-	-

**Table 3:** Pixel correlation between 1000 random pixels of Lena.

Algorithm	Horizontal	Vertical	Diagonal
[47]	−0.0098	−0.0050	−0.0013
[50]	−0.0023	0.0019	−0.0034
[51]	0.0020	−0.0007	−0.0014
[48]	−0.0237	−0.0178	−0.0284
[49]	0.0080	0.0098	−0.0058
Proposed	−0.0042707	−0.0032498	−0.020192

**Table 4:** Pixel correlation of USC−SIPI.

Images	Channel	Plain			Ciphered		
		Horizontal	Vertical	Diagonal	Horizontal	Vertical	Diagonal
4.1.01	Red	0.97318	0.96395	0.94653	−0.0053517	0.010961	−0.0049967
	Green	0.9692	0.96409	0.94567	−0.0047375	−0.0012887	0.0059078
	Blue	0.95666	0.94848	0.93846	0.0021301	0.0023748	−0.00036386
4.1.02	Red	0.94829	0.95259	0.91853	−0.00331	−0.0041589	0.016235
	Green	0.9237	0.95515	0.89936	−0.012505	−0.00032095	0.0032421
	Blue	0.91566	0.94324	0.88315	0.018044	−0.00053454	0.019847
4.1.03	Red	0.98061	0.92609	0.90982	−0.01801	−0.012732	−0.01593
	Green	0.97722	0.91153	0.88157	0.0009692	0.0024241	0.0076471
	Blue	0.97383	0.90929	0.89348	−0.0072574	0.0084441	0.0036835
4.1.04	Red	0.97653	0.98664	0.97151	−0.0063601	0.021812	−0.020985
	Green	0.96491	0.98351	0.95113	−0.0085227	−0.01142	−0.0089817
	Blue	0.95215	0.97005	0.93335	0.0018656	0.0041814	−0.011218
4.1.05	Red	0.96786	0.93611	0.91023	0.0025253	−0.0070104	−0.001377
	Green	0.98024	0.95152	0.92792	0.0015667	−0.013295	0.0082844
	Blue	0.98215	0.97459	0.96133	−0.0077072	−0.0074648	−0.0048926
4.1.06	Red	0.95813	0.93145	0.91129	−0.0092083	−0.01433	0.0011584
	Green	0.96761	0.94832	0.92968	−0.0020563	0.0052499	0.0073594
	Blue	0.96175	0.94106	0.92656	0.00096206	−0.0032515	0.013931
4.1.07	Red	0.97582	0.97547	0.94929	0.0032059	−0.0079411	−0.0082824
	Green	0.9777	0.98171	0.95789	−0.0037171	−0.0054693	0.0013666
	Blue	0.98938	0.98803	0.97825	−0.010115	0.00021278	−0.00074795
4.1.08	Red	0.97278	0.97356	0.95062	−0.0035631	−0.0040268	0.004974
	Green	0.97332	0.97477	0.94604	−0.002521	−0.0092897	−0.0079288
	Blue	0.97943	0.98097	0.95977	0.0090987	−0.0020318	−0.0036913
Apricot	Red	0.98386	0.9863	0.96945	−0.013663	−0.0018757	−0.0054083
	Green	0.97884	0.98512	0.96538	−0.010663	0.0015055	0.0023429
	Blue	0.98725	0.99154	0.98349	0.0048932	−0.0057104	−0.011769
Vegetables	Red	0.97887	0.98013	0.96109	0.001243	−0.0032611	0.00075363
	Green	0.9775	0.97953	0.95948	−0.00075568	−0.0024519	0.0044758
	Blue	0.97225	0.97092	0.9473	−0.0010255	−0.0049976	0.0015237
Fruits	Red	0.9868	0.98516	0.97407	−0.0044958	−0.0014318	0.0047764
	Green	0.98084	0.98078	0.96405	−0.0057875	−0.00091906	−0.0066637
	Blue	0.95394	0.9494	0.90847	−0.0040615	0.0012303	−0.005508
baby	Red	0.97834	0.97816	0.96278	−0.006159	0.0021686	0.0025641
	Green	0.99107	0.99428	0.97854	−0.0021032	0.0011388	0.0099095
	Blue	0.99371	0.99472	0.98814	−0.0036711	−0.012494	0.0046702
Panda	Red	0.95176	0.96553	0.93162	0.005131	−0.00076785	−0.0049536
	Green	0.95216	0.96437	0.93067	0.0078787	−0.00079504	0.00027378
	Blue	0.95543	0.97087	0.94266	$7.0511\times 10^{-5}$	−0.010969	0.01059
Nike	Red	0.98682	0.9922	0.97288	−0.0043816	−0.016514	0.0066848
	Green	0.98852	0.99104	0.97353	0.0023924	0.0017956	−0.0034512
	Blue	0.98676	0.9905	0.9719	0.0087522	0.0057947	−0.0030273
Tiffany	Red	0.9584	0.95431	0.91675	−0.0014434	−0.0019299	0.0075856
	Green	0.89597	0.95016	0.87161	−0.012698	0.0075576	−0.001533
	Blue	0.91305	0.93013	0.86384	−0.0014111	0.0010208	−0.0038235

**Table 5:** Number of pixels change rate (NPCR) and unified average changing intensity (UACI) comparison.

Algorithm	Lena		Pepper	
	NPCR_R,G,B_	UACI_R,G,B_	NPCR_R,G,B_	UACI_R,G,B_
**Proposed**	0.9968	0.3346	0.9967	0.3348
[52]	0.9966	0.3344	0.9963	0.3347
[53]	0.9959	0.3346	-	-
[56]	0.9962	0.3365	-	-
[57]	0.9960	0.3348	-	-
[54]	0.9960	0.3344	-	-
[55]	0.9961	0.3346	0.9960	0.3349

**Table 6:** Known/chosen plain-text attack result.

Algorithms	Images	Entropy	Correlation			NPCR	UACI
			Horizontal	Vertical	Diagonal	SW,SB	SW,SB
Ours	All white	0	-	-	-	-	-
	Cipher white	7.9988	−0.01140	−0.00706	0.00631	0.9962	0.3349
	Full black	0	-	-	-	-	-
	Cipher black	7.9989	0.00965	−0.00307	−0.00172	0.9959	0.3343
[63]	All white	0	-	-	-	-	-
	Cipher white	7.9971	0.0071	0.0144	−0.0068	-	-
	Full black	0	-	-	-	-	-
	Cipher black	7.9970	−0.0101	0.0135	−0.0010	-	-

**Table 7:** $E_{Q}$ comparison of USC-SIPI database.

USC-SIPI	Proposed			[64]		
	Red	Green	Blue	Red	Green	Blue
4.1.01	358	385	292	312	317	326
4.1.02	229	289	272	336	347	346
4.1.03	393	370	403	308	346	339
4.1.04	272	210	284	203	199	282
4.1.05	219	278	231	290	273	312
4.1.06	210	252	260	189	181	246
4.1.07	285	289	244	372	358	291
4.1.08	242	264	261	331	309	259
House	284	317	276	183	219	155

**Table 8:** Peak signal to noise ratio (PSNR) (dB) between plain (P) ↦ cipher (C) image and plain ↦ decrypted (D) image.

Algorithm	PSNR type	Lena	Baboon	Panda	Vegetables
**Proposed**	P to D	∞	∞	∞	∞
	P to C	8.1102	8.7776	8.1648	6.8760
[66]	P to C	8.1300	7.8569	7.7410	7.4395
[65]	P to D	96.295	-	-	-
	P to C	9.0348	-	-	-
[67]	P to C	8.3655	8.8532	-	-
[69]	P to C	8.2522	8.8223	-	-

**Table 9:** PSNR (dB) and mean square error (MSE) comparison with different cropped size.

Cropped Size	Image	Proposed		[68]		[64]	
		PSNR	MSE	PSNR	MSE	PSNR	MSE
1/2	Lena	17.4286	$1.1755\times 10^{3}$	12.88	3121.1	11.58	4578.34
	Panda	13.9065	$2.6450\times 10^{3}$	-	-	-	-
1/4	Lena	20.2816	609.4230	14.722	2192.2	14.59	2289.9
	Panda	16.7175	$1.3846\times 10^{3}$	-	-	-	-
1/8	Lena	23.0749	320.3273	16.75	1375.9	17.57	1155.3
	Panda	19.4912	731.0670	-	-	-	-
1/16	Lena	25.6379	177.5376	19.25	772.65	20.57	579.9
	Panda	21.9180	418.0993	-	-	-	-

**Table 10:** Information entropy comparison.

Algorithm	Test	Ciphered		
	Image	R	G	B
**Our**	Lena	7.9974	7.9974	7.9971
	Pepper	7.9993	7.9994	7.9992
	Baboon	7.9993	7.9992	7.9993
	Panda	7.9972	7.9971	7.9966
	Vegetable	7.9992	7.9994	7.9994
[58]	Lena	7.989	7.989	7.989
	Pepper	7.989	7.988	7.989
	Baboon	7.989	7.989	7.988
	Panda	7.988	7.989	7.989
	Vegetable	7.989	7.989	7.989
[62]	Lena	7.987	7.987	7.986
[70]	Lena	7.927	7.974	7.970
[71]	Lena	7.973	7.975	7.971
[10]	Lena	7.987	7.988	7.987

**Table 11:** Global entropy and Shannon entropy.

Images	Our	${\mathrm{Global}}_{R,G,B}$		[73]
	Shannon_C_	Plain	Cipher	Shannon_C_
Vegetables	7.9032	7.5060	7.9997	-
Baby	7.9029	6.3988	7.9997	-
Fruits	7.9023	7.5322	7.9991	-
Panda	7.9026	7.8679	7.9990	-
Pepper	7.9030	7.6698	7.9998	7.9024
Apricot	7.9029	5.7492	7.9997	-
Nike	7.9022	1.1969	7.9997	
Tiffany	7.9023	6.4288	7.9998	-
House	7.9032	7.4858	7.9997	7.9021
Baboon	7.9027	7.2428	7.9998	7.9023
Lena	7.9032	7.4517	7.9991	7.9024
4.1.01	7.9023	6.8981	7.9991	-
4.1.02	7.9032	6.2945	7.9990	-
4.1.03	7.9019	5.9709	7.9991	
4.1.04	7.9021	7.4270	7.9992	-
4.1.05	7.9033	7.0686	7.9990	-
4.1.06	7.9031	7.5371	7.9991	-
4.1.07	7.9020	6.5835	7.9989	-
4.1.08	7.9027	6.8527	7.9991	-

**Table 12:** Gray value difference comparison.

USC-SIPI	Proposed			[64]			[74]			[8]		
	GVD			GVD			GVD			GVD		
	Red	Green	Blue	Red	Green	Blue	Red	Green	Blue	Red	Green	Blue
4.1.01	0.995	0.943	0.996	0.977	0.979	0.975	-	-	-	-	-	-
4.1.02	0.970	0.999	0.999	0.978	0.979	0.979	-	-	-	-	-	
4.1.03	0.672	0.407	0.408	0.978	0.976	0.977	-	-	-	-	-	-
4.1.04	0.383	0.348	0.532	0.979	0.975	0.980	-	-	-	-	-	-
4.1.05	0.673	0.918	0.719	0.982	0.966	0.969	-	-	-	-	-	-
4.1.06	0.988	0.995	0.989	0.943	0.912	0.934	-	-	-	-	-	-
4.1.07	0.245	0.382	0.794	-	-	-	-	-	-	-	-	-
4.1.08	0.564	0.355	0.291	0.985	0.973	0.983	-	-	-	-	-	-
4.2.01	0.782	0.804	0.905	-	-	-	-	-	-	-	-	-
4.2.03	0.998	0.985	0.986	-	-	-	-	-	-	0.9801	0.989	0.9865
4.2.05	0.934	0.460	0.565	-	-	-	-	-	-	-	-	-
4.2.06	0.453	0.261	0.879	-	-	-	-	-	-	-	-	-
4.2.07	0.511	0.329	0.571	-	-	-	-	-	-	-	-	-
Lena	0.979	0.997	0.995	-	-	-	0.980	0.981	0.987	0.970	0.970	0.969

**Table 13:** Encryption time (in seconds) comparison.

Image Size	Proposed	[75]	[76]	[77]
256×256	**2.09**	4.7795	3.617	4.221
512×512	**3.77**	8.670	14.811	21.178

## Supplementary Materials

The following are available online at https://www.mdpi.com/1099-4300/22/1/112/s1.
